# A practical guide to EEG hyperscanning in joint action research: from motivation to implementation

**DOI:** 10.1093/scan/nsae026

**Published:** 2024-04-03

**Authors:** Anna Zamm, Janeen D Loehr, Cordula Vesper, Ivana Konvalinka, Simon L Kappel, Ole A Heggli, Peter Vuust, Peter E Keller

**Affiliations:** Department of Linguistics, Cognitive Science and Semiotics, Aarhus University, Aarhus 8000, Denmark; Interacting Minds Center, Aarhus University, Aarhus 8000, Denmark; Department of Psychology and Health Studies, University of Saskatchewan, Saskatoon, SK S7N 5A5, Canada; Department of Linguistics, Cognitive Science and Semiotics, Aarhus University, Aarhus 8000, Denmark; Interacting Minds Center, Aarhus University, Aarhus 8000, Denmark; Section for Cognitive Systems, DTU Compute, Technical University of Denmark, Kongens Lyngby DK-2800, Denmark; Department of Electrical and Computer Engineering, Aarhus University, Aarhus N 8200, Denmark; Center for Music in the Brain, Department of Clinical Medicine, Aarhus University & The Royal Academy of Music Aarhus/Aalborg, Aarhus 8000, Denmark; Center for Music in the Brain, Department of Clinical Medicine, Aarhus University & The Royal Academy of Music Aarhus/Aalborg, Aarhus 8000, Denmark; Center for Music in the Brain, Department of Clinical Medicine, Aarhus University & The Royal Academy of Music Aarhus/Aalborg, Aarhus 8000, Denmark; MARCS Institute for Brain, Behaviour and Development, Western Sydney University, Penrith, New South Wales 2751, Australia

**Keywords:** EEG hyperscanning, joint action, social neuroscience, reproducibility, research methods

## Abstract

Developments in cognitive neuroscience have led to the emergence of hyperscanning, the simultaneous measurement of brain activity from multiple people. Hyperscanning is useful for investigating social cognition, including joint action, because of its ability to capture neural processes that occur within and between people as they coordinate actions toward a shared goal. Here, we provide a practical guide for researchers considering using hyperscanning to study joint action and seeking to avoid frequently raised concerns from hyperscanning skeptics. We focus specifically on Electroencephalography (EEG) hyperscanning, which is widely available and optimally suited for capturing fine-grained temporal dynamics of action coordination. Our guidelines cover questions that are likely to arise when planning a hyperscanning project, ranging from whether hyperscanning is appropriate for answering one’s research questions to considerations for study design, dependent variable selection, data analysis and visualization. By following clear guidelines that facilitate careful consideration of the theoretical implications of research design choices and other methodological decisions, joint action researchers can mitigate interpretability issues and maximize the benefits of hyperscanning paradigms.

## Introduction

Hyperscanning, the simultaneous measurement of brain activity from multiple people (Montague *et al*., 2002), has become an increasingly popular method in cognitive neuroscience and beyond. Hyperscanning holds appeal for researchers who study joint action, i.e. the coordination of actions across multiple individuals towards a shared goal (Sebanz *et al*., 2006), because it can address research questions regarding neural processes that happen not only ‘within’ individual group members (i.e. intra-brain) but also ‘across’ group members (inter-brain; Dumas *et al*., 2011; Konvalinka and Roepstorff, 2012). At the same time, however, skeptical voices have raised questions about the utility of hyperscanning, based on concerns regarding the interpretability of inter-brain measures and lack of a unifying theoretical approach (Hamilton, 2021; Holroyd, 2022). These contrasting viewpoints can leave joint action researchers both interested in and hesitant about including hyperscanning in their research programs, wondering, ‘Can hyperscanning help answer my research questions? What pitfalls do I need to watch out for, and how can these be addressed as I design and carry out a hyperscanning project?’. The immediate purpose of this guide is to provide researchers with key considerations toward answering such questions when planning and implementing hyperscanning studies. Our broader aims are 2-fold: first, to reduce the likelihood that researchers will confront interpretability pitfalls pointed out by hyperscanning skeptics after they have already collected and analyzed their data, and second, to support the move toward standardizing methodological approaches in hyperscanning, particularly within joint action research (Ayrolles *et al*., 2021).

We have organized the guide in a style akin to a ‘frequently asked questions’ list. We pose questions likely to be encountered by our intended audience: joint action researchers who are considering whether and how to dive into hyperscanning because it might augment their ability to answer meaningful research questions about social cognition and interaction. We also hope the guide will be useful for researchers and scholars in other disciplines, such as fine arts, social sciences and engineering, with whom we often find ourselves conversing about the utility and complexity of hyperscanning, in particular for capturing joint action phenomena. This guide is meant to be used to steer decision-making about how to best plan and implement hyperscanning studies. It is not intended to be a technical manual or a perspectives paper—we refer readers to other sources for this.

Because our guide is aimed primarily toward joint action researchers, we focus specifically on EEG hyperscanning—which uses electroencephalography to record cortical electrical signals from multiple individuals—because it is optimally suited for capturing the millisecond-level dynamics of joint action coordination and is widely used in the joint action community. That said, many of the questions and considerations we describe are broadly applicable across neuroimaging modalities and academic disciplines. For example, general issues about study conceptualization and experiment design are equally applicable in hyperscanning studies that use techniques such as functional magnetic resonance imaging (fMRI; Misaki *et al*., 2021) and functional near-infrared spectroscopy (fNIRS; Scholkmann *et al*., 2013) while technical details about data acquisition and analysis are specific to EEG.

The first question addressed in the guide is whether hyperscanning would be a necessary and valid choice to answer one’s joint action research questions (Section ‘Do I need hyperscanning to answer my joint action research question?’). The remaining questions concern decisions that need to be made once a researcher has decided to undertake a hyperscanning project, including study design, selection of dependent variables, data analysis and data visualization (Sections ‘What are key experiment design considerations for hyperscanning studies?’, ‘Hyperscanning projects can yield intimidating datasets. How do I home in on the dependent variable(s) that are best suited to answer my research question?’ and ‘What issues will I need to consider as I progress through the stages of analyzing my hyperscanning data?’). Table 1 summarizes the questions posed in each section and key considerations with respect to answering them. To set the stage for these questions, we first briefly review existing literature that describes the utility of hyperscanning (Section ‘Why are joint action researchers so excited about hyperscanning?’) as well as literature that has raised concerns about hyperscanning (Section ‘What concerns have been raised about hyperscanning?’).

**Table 1.** Key questions and considerations when implementing a hyperscanning study

Section	Question	Considerations
2	Do I need hyperscanning to answer my research question?	Some research questions can only be addressed by examining inter-brain data. Others can be addressed by examining intra-brain or behavioral data, both of which can be collected in hyperscanning set-ups. Still others might require brain stimulation or computational modeling techniques. Broad research questions are likely best answered by designing studies that, iteratively and recursively, address each of the above options as well as the connections between them.
3	What are key experiment design considerations for hyperscanning studies?	Broad research questions are likely best answered by designing studies that, iteratively and recursively, capitalize on the complementary methodological strengths of lab-based and real-world approaches. Careful experiment designs can mitigate concerns regarding whether inter-brain coupling is driven by exogenous or endogenous sources. Key goals in hyperscanning experiment design are to ensure that conditions allow for capturing baseline levels of inter-brain coupling between partners performing identical actions individually, to minimize signal-to-noise ratio differences between conditions and to minimize temporally aligned noise coupling between participants.
4	How do I select the dependent variable(s) best suited to answer my research question?	Inter-brain measures (including phase synchrony, envelope coupling, wavelet coherence and directional measures) can be selected based on their suitability for a given research question. Solo action and intra-brain studies can provide a useful starting point for selecting which EEG frequency bands and spatial locations to analyze. Time windows for analysis can take into consideration behaviorally meaningful intervals, signal stationarity and the number of oscillation cycles required to capture patterns of interest.
5	What issues do I need to consider as I progress through the stages of analyzing my hyperscanning data?	Data inspection steps include checking behavioral data for expected patterns across experimental conditions and examining intra-brain data to ensure neural oscillations of interest are present. When selecting a statistical analysis strategy, researchers may need to consider the multi-level (nested) structure of joint action data, how to define ‘chance’ for permutation-based analyses and options for data-driven approaches to analysis. When presenting the results of a hyperscanning study, data visualizations benefit from including preprocessing steps and spatiotemporal visualizations of intra- and inter-brain signals, in addition to aggregated statistical results.

### Why are joint action researchers so excited about hyperscanning?

In the past decade, there has been much excitement about hyperscanning and a burst of new research employing hyperscanning techniques. Recent review articles cover this literature from various perspectives. Several reviews have addressed the range of neuroimaging techniques that are used for hyperscanning (including EEG, fNIRS and fMRI) and delineated the insights each approach has revealed, including for joint action (e.g. Babiloni and Astolfi, 2014; Wang *et al*., 2018; Czeszumski *et al*., 2020). Other reviews have focused specifically on EEG hyperscanning, outlining, for example, the use of hyperscanning to investigate continuous interactions in contexts of high ecological validity (Acquadro *et al*., 2016) or the processes underlying cooperative *vs* competitive tasks (Balconi and Vanutelli, 2017). Still other reviews highlight specific applications of hyperscanning, e.g. to creativity and the performing arts (e.g. Shemyakina and Nagornova, 2021) and parent–child interactions (Hoehl and Bertenthal, 2021; Turk *et al*., 2022).

Hyperscanning is particularly exciting for joint action researchers because measuring multiple people’s brain activity and examining the ‘relations between’ people’s brain activity allows researchers to capture the fundamental interconnectedness between co-actors’ cognitive and neural processes that occur during joint action. When two or more people coordinate their actions to achieve a joint goal, their action planning and execution become closely linked (see, e.g. Vesper *et al*., 2010; Knoblich *et al*., 2011; Keller *et al*., 2016; Pesquita *et al*., 2018; Müller *et al*., 2021). For example, pair dancers fine-tune their movements to each other to glide in perfect synchrony along the dance floor; jazz musicians monitor their own contribution to the overall musical sound and influence it continuously through their own playing; a couple solving crossword puzzles uses verbal communication to inspire each other to new ways of thinking, relying closely on the complementarity of their expertise. Focusing on the cognitive and neural processes of each individual separately might not fully capture the interconnectedness created by these continuous mutual influences between joint action partners, where each person’s actions have direct consequences for ther other person(s) and the shared environment (Konvalinka and Roepstorff, 2012). Instead, additional insights are gained by examining group-level processes, including coupling between partners’ neural activity. Indeed, some theories go so far as to claim that joint action is generally not reducible to individual processes and that any individual processes can therefore only be understood in relation to group-level processes (Marsh *et al*., 2009).

Although we do not aim to review or evaluate existing theories of joint action here, it can be noted that different theoretical perspectives address individual- and group-level processes to varying degrees. Co-representational accounts often can be tested sufficiently at the individual level (Loehr *et al*., 2013; Novembre *et al*., 2016), dynamical systems hypotheses may necessitate testing at the group level (Dumas *et al*., 2010; Zamm *et al*., 2021) and predictive coding accounts focus on both levels to the extent that top-down processes (e.g. expectations based on prior experience) can be indexed at the individual level while bottom-up processes (e.g. convergence of shared expectations via mutual adaptation) can be assessed at the group level (Vuust *et al*., 2022). Given such considerations, ‘using hyperscanning to investigate the neural processes that occur both within and between brains during joint action can further our goal to fully understand its cognitive and neural bases’.

### What concerns have been raised about hyperscanning?

Alongside literature documenting the insights gained through hyperscanning, researchers have also argued that caution is warranted because the technological and interpretational challenges it poses are not trivial. Some researchers have voiced concern over the inherently correlational nature of hyperscanning and argued that it might have little utility for understanding causal mechanisms of social interaction (Novembre and Iannetti, 2021b). Others have raised concerns about the field’s lack of a coherent theoretical framework for investigating inter-brain coupling, as well as the heterogeneity of inter-brain measurement methods (Holroyd, 2022). Concerns have also been raised that any inter-brain coupling captured by hyperscanning risks being merely an epiphenomenon of shared sensorimotor input between social interaction partners (Burgess, 2013; Hamilton, 2021; Holroyd, 2022).

This latter point—whether inter-brain coupling is epiphenomenal—is particularly important for joint action researchers. Specifically, this point reflects a central debate over whether coupling between partners’ brain activity can fully be accounted for by each partner’s exposure to the same sensory information or can instead reflect higher-level processes. In other words, some effects of inter-brain coupling might be driven by stimuli that are present in the environment (‘exogenous sources’), such as hearing the same sounds or seeing the same images (e.g. Hasson *et al*., 2004; Pérez *et al*., 2021; Madsen *et al*., 2022). In other cases, effects of inter-brain coupling might capture aligned action plans, common background knowledge or social belonging processes (‘endogenous sources’). Exogenous and endogenous sources of information can produce identical effects on behavior and brain activity. As a simple example, two musicians might play a piece at 90 beats-per-minute because they are both playing along with the same metronome (exogenous) or because the two individuals have previously agreed on an appropriate tempo for their joint playing (endogenous). This is in itself not a problem, as either case can be of interest for understanding joint action. However, as pointed out (e.g. by Holroyd, 2022), it can become a problem if researchers are not careful about what they measure and how they interpret their results. In line with this, Hamilton (2021) points out that inter-brain correspondences are often over-interpreted as demonstrating high-level social phenomena such as empathy or affiliation, when they might reflect common motor or cognitive processing of shared stimuli.

‘Critically, the above concerns can be mitigated in part through careful experiment design, operational definitions of measurement variables and data analysis and visualization strategies’. We delineate a number of these considerations in Sections ‘What are key experiment design considerations for hyperscanning studies?’, ‘Hyperscanning projects can yield intimidating datasets. How do I home in on the dependent variable(s) that are best suited to answer my research question?’ and ‘What issues will I need to consider as I progress through the stages of analyzing my hyperscanning data?’. We also refer the reader to recent efforts to create standardized methodological choices (Zimmerman et al., 2023) and data analysis pipelines (Ayrolles *et al*., 2021; Kayhan *et al*., 2022), which are also critical steps in addressing these concerns.

Having now provided a general overview of the benefits of hyperscanning as well as reasons to be cautious in embracing it, we turn in the next section to the first question researchers might ask themselves if they feel excited about hyperscanning, namely, ‘do I need hyperscanning to answer my joint action research question?’.

## Do I need hyperscanning to answer my joint action research question?

In this section, we focus on hyperscanning’s utility for answering different kinds of research questions in joint action. We begin by suggesting that researchers who are contemplating adding hyperscanning to their research program first consider which level(s) of explanation their research questions seek to address. Classic approaches to levels of understanding in cognitive (neuro)science have highlighted the benefits of considering multiple levels (e.g. Pylyshyn, 1984; Anderson, 1990; Marr, 2010; Acquadro *et al*., 2016). Concretely, these levels deal with (i) the goals of the system or of a particular computational process that the system performs (‘why’), (ii) the executive processes or algorithms used to realize these goals (‘what’) and (iii) the biological mechanisms by which these processes are implemented (‘how’). Questions pitched at the levels of ‘why’ and ‘what’ can be answered by analyzing behavior, while questions about ‘how’ lead to the analysis of brain structure and function (Krakauer *et al*., 2017). Critically, answers at one level inform and constrain hypotheses and explanations at other levels (Marr, 2010).

**Fig. 1.**
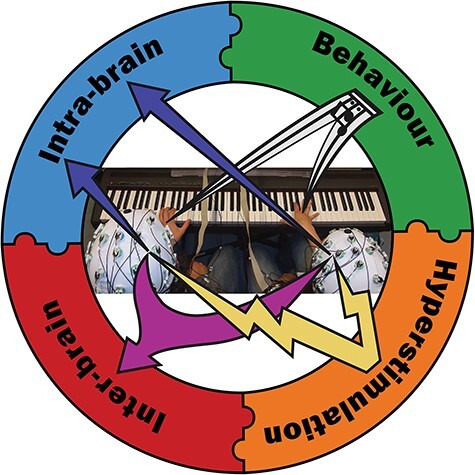
Broad research questions can be answered by triangulating across levels of analysis (inter-brain, intra-brain and behavioral data) and complementary techniques (hyperstimulation).

Hyperscanning paradigms obviously involve analyses of neural signals (‘how’), usually to inform understanding of behavior and cognition (‘what’ and ‘why’), e.g. behaviors such as interpersonal synchrony and turn-taking in conversation, and cognitive functions such as joint attention and prediction. Importantly, the term ‘hyperscanning’ is strongly associated with examining neural signals at the group level, i.e. with examining the relations between multiple people’s brain activity, hereafter referred to as ‘inter-brain data’ (and elsewhere variously referred to as inter-brain synchronization, inter-brain coupling, inter-brain coordination, etc.). Indeed, the reviews discussed in Section ‘Why are joint action researchers so excited about hyperscanning?’ focus almost exclusively on the insights gained by examining inter-brain data. However, hyperscanning paradigms do not ‘only’ offer the possibility of examining inter-brain data (as illustrated in Figure 1). They can also be used to examine neural signals at the individual level, i.e. to examine individual people’s brain activity, hereafter referred to as ‘intra-brain data’. Furthermore, ‘behavioral data’, which offer the possibility of addressing links between brain and behavior, are also often (but not always) acquired during hyperscanning studies. In the sections that follow, we briefly outline when and why one’s research questions would require inter-brain, intra-brain and/or behavioral data (Sections 2.1–2.3). We also briefly describe how interpretation of these three types of data can be aided by complementary techniques such as brain stimulation (Section 2.4), and how interrelations between inter-brain, intra-brain and behavioral data and between hyperscanning and stimulation techniques can be addressed (Section 2.5).

### Does my research question require inter-brain data?

Inter-brain measures can be used to determine whether partners display corresponding or coupled patterns of cortical activity during joint action. Inter-brain measures are ‘necessary’ when the research question seeks to address the relations between partners’ brain activity, such as the strength of coupling between partners or the directionality of coupling (e.g. whether one partner’s brain activity leads or follows the other’s). Furthermore, joint actions typically span multiple modalities (visual, auditory, haptic, etc.), and therefore shape not only the behavior of the interacting participants but also their shared environment. Accordingly, inter-brain measures can capture elements of the interaction that cannot be readily measured by examining behavior alone (e.g. communicative or intentional alignment between partners). Inter-brain measures can therefore provide additional insight into the mechanisms that underlie higher-level processes in joint action, such as representations of joint goals or communication between partners, that intra-brain or behavioral data alone cannot provide. For example, studies of joint music performance have used inter-brain coupling measures to assess alignment of performers’ internal timekeeping, which may not always be consistent with externally measured behavior (e.g. Gugnowska *et al*., 2022).

In addition, researchers can choose inter-brain measures for practical reasons. For example, discrete behaviors are often underpinned by continuous neural processes, such as in conversational turn-taking, where each turn is discrete, but speech planning and monitoring are continuous processes that span across individual turns (Bögels and Levinson, 2017). In such cases, continuous neural measurement from partners during ongoing interaction affords insight into cognitive processes that cannot be acquired from measuring behavior alone. Indeed, one important reason why hyperscanning studies have become increasingly popular is that they allow researchers to sample data from multiple people continuously and in alignment.

### Can my research question be answered with intra-brain data?

Some joint action research questions address individual-level brain mechanisms and thus can be answered by using hyperscanning to collect intra-brain data. For example, researchers might be interested in investigating individual action planning processes involved in achieving joint goals or examining how social emotions such as affiliation or interpersonal relations such as group membership influence each individual partner’s neural response to the other’s actions. Hyperscanning can be useful in such contexts because one can collect neural data from the same pair or group of participants engaged in the same joint action (which also has the practical advantage of collecting multiple data sets within one session). As an example, Loehr *et al*. (2013) studied goal monitoring processes in individual brains of two simultaneously recorded pianists performing a musical joint action. Their findings revealed that violations of a joint musical goal modulated individual performers’ event-related potentials (ERPs), demonstrating that co-actors represented and monitored their individual actions in relation to the joint goal. Intra-brain analyses were sufficient because the research question did not target inter-brain processes, but instead targeted answers about individual performers’ action monitoring in relation to a joint goal.

### How does behavioral data factor into the research question?

In many cases, researchers are keenly interested in the dynamics of behavior associated with brain activity, and therefore it is important to take behavioral measures seriously in any hyperscanning paradigm. Indeed, a growing chorus of influential cognitive neuroscientists has been advocating for the study of behavioral questions as a matter of priority. The crux of the argument is that the same behavior can be produced by different neural processes and hence examining the brain will not alone explain how it generates behavior (Gazzaniga, 2010; Krakauer *et al*., 2017). The importance of examining behavior alongside brain activity specifically within the context of hyperscanning has also recently been emphasized by Hamilton (2021). One example of how behavioral measures can be critical for interpreting hyperscanning data is laid out by Schoot *et al*. (2016), who discuss the situation of speaker(s) and listener(s) engaged in a conversation, where neither’s actions are meaningful outside of the social interaction. As these authors point out, detecting correlated activity between speakers’ and listeners’ brains is not sufficient to draw conclusions about the mechanisms by which they coordinate. Instead, the quality of each specific interaction needs to be taken into account to ensure that valid inferences are drawn about whether inter-brain correlations provide a neural foundation for communication success.

We note here that the behavioral level of analysis is a legitimate level all on its own. For example, researchers have argued that clever experimental designs can reveal functional principles from behavior alone (Rosenbaum, 2005) and that factorial designs that allow additive and interactive factors to be discerned can provide access to the architectural building blocks of human cognition from even the minimal behavior of a button press (Sternberg, 2001, 2011). In other words, some research questions can be answered with a behavioral study and, in that case, hyperscanning might not be needed.

### How do direct manipulations of brain and behavior complement hyperscanning?

Often, researchers are interested in how correspondences between partners’ brain activity are causally related to their coordination of joint actions. In these cases, it can be useful to not only measure brain activity and behavior but also to directly manipulate them. Direct manipulations of brain and behavior are possible through non-invasive brain stimulation, which can be implemented by experimental manipulations and/or direct electromagnetic stimulation techniques such as transcranial magnetic stimulation (TMS) or transcranial alternating current stimulation (tACS). The latter is referred to as multi-brain or hyper-stimulation when applied to two or more brains simultaneously (Pan *et al*., 2021; Novembre and Iannetti, 2021a). In the case of stimulation through experimental manipulation, participants are presented with carefully selected sensory stimuli, a method that is the bread and butter of experimental psychology, but which is relatively indirect (i.e. the experimenter can control the sensory regions that are activated by presenting modality-specific stimuli, but not necessarily other regions). In contrast, electromagnetic stimulation allows specific brain regions to be targeted more directly, albeit with low levels of spatial resolution and not with uniform coverage. Stimulation techniques can be used to increase or inhibit neural excitability in particular brain regions or circuits, and thereby causally manipulate specific neural processes (Polanía *et al*., 2018; Reed and Cohen Kadosh, 2018). One example of a study that used hyper-stimulation to study the causal effects of inter-brain coupling on joint action coordination was carried out by Novembre *et al*. (2017), who used tACS during a joint finger tapping task. By modulating neural oscillations in individual members of a pair, they provided evidence that frequency- and phase-specific patterns of inter-brain synchrony facilitate people’s ability to generate predictions about each other’s timing and thereby coordinate with each other.

### How can we integrate findings across levels of analysis?

Combining different types of data (inter-brain, intra-brain, behavioral) and techniques (hyperscanning, hyperstimulation) can maximize the interpretability of obtained study results and address connections between different levels of analysis (Krakauer *et al*. (2017). As an example, the stimulation frequencies and locations of the Novembre *et al.’s* (2017) study described in Section ‘How do direct manipulations of brain and behavior complement hyperscanning?’ were selected based on a body of previous work examining rhythmic coordination at the behavioral, intra-brain and inter-brain levels. Another option is to examine connections between levels within a single study. One example of this strategy is provided in the Schoot *et al.’s* (2016) study described in Section ‘How does behavioral data factor into the research question?’, where behavior and brain correlates were equally important for answering the research question. Other examples include adding group as a factor in the statistical analysis of intra-brain data to estimate how group-level characteristics influence individual brain measures (e.g. Kenny *et al*., 2020; see also Section ‘Do my statistical models capture the multi-level structure of joint action data?’) or conducting intra-brain analyses to ensure that neural responses of interest are present within each member of a group before examining inter-brain correspondence of those responses (e.g. A. Zamm *et al*., 2021; see Section ‘What should I consider when initially inspecting the data?’). Finally, computational modeling can be a useful tool for examining connections across levels, including linking behavioral patterns to brain activity and mapping functional descriptions (e.g. at an algorithmic level) to explanatory mechanisms (at a physiological level) (Forstmann and Wagenmakers, 2015; Frank and Badre, 2015). More specifically, computational models require researchers to quantitatively describe reciprocal interactions between brain and behavior during joint action.

Ultimately, our view is that broad research questions are likely best answered by triangulating findings across all levels of analysis and across complementary methodological techniques, as illustrated in Figure 1. Joint actions are complex and dynamic, and therefore understanding them often requires focusing on specific levels individually, identifying clear patterns at each level and subsequently integrating across levels, both within and across studies. Thus, we would encourage researchers to, individually or collectively, design studies that iteratively and recursively address each level of analysis and the connections between them.

Next, we turn to the questions researchers will encounter once they have decided to undertake a hyperscanning study (Sections ‘What are key experiment design considerations for hyperscanning studies?’, ‘Hyperscanning projects can yield intimidating datasets. How do I home in on the dependent variable(s) that are best suited to answer my research question?’ and ‘What issues will I need to consider as I progress through the stages of analyzing my hyperscanning data?’). Some questions and answers pertain specifically to studies that focus on inter-brain data, but others are applicable regardless of the level of data being analyzed.

## What are key experiment design considerations for hyperscanning studies?

Once a researcher has decided to undertake a hyperscanning study, they will need to make choices related to experimental design. Here we outline several key questions that will need to be answered, including in which context to run the experiment, which conditions to include in the experiment and additional practical considerations.

### Should I carry out my studies in the lab or the ‘real world’?

Researchers have long discussed the relative benefits and drawbacks of laboratory-based approaches, in which experimenters isolate and systematically manipulate one or a few variables while attempting to hold all other variables constant, and ‘real-world’ approaches, in which behavior and brain activity are examined under naturalistic conditions that capture the complexity and richness of everyday settings (see e.g. ). Lab-based approaches have been historically dominant in cognitive psychology and cognitive neuroscience, but recently there has been a shift toward embracing real-world settings (e.g. Barthel *et al*., 2017; Matusz *et al*., 2019; Shamay-Tsoory and Mendelsohn, 2019; Nastase *et al*., 2020; Dikker *et al*., 2021). For example, Dikker and colleagues (Dikker *et al*., 2021) demonstrated how hyperscanning techniques can be taken out of the lab and applied in real-world settings. They undertook a large-scale effort to crowdsource neuroscience data of face-to-face social interactions by measuring EEG data from thousands of art museum visitors over a period of 5 years, showing that visitors’ social behavior, social closeness and certain personality traits were associated with inter-brain synchronization across alpha and beta oscillations.

More often than not, lab-based and real-world approaches can be considered to be complementary, making it valuable to examine research questions iteratively and recursively from both perspectives to gain a full understanding of a phenomenon and its underlying mechanisms (Dhami *et al*., 2004; Kingstone *et al*., 2008; Holleman *et al*., 2020). As an example, G. Novembre, D. Sammler and P. Keller *et al*. (2016) first established that centro-parietal alpha oscillations reflect self-other integration and distinction during joint music making in a lab-based study with pairs of duetting pianists, and Christensen *et al*. (2023) subsequently demonstrated that such oscillations are likewise associated with self-other integration and distinction among violinists performing a repertoire piece within a 60-musician orchestra. Regardless of whether implementing real-world or lab-based experiments, it is important to consider how variables of interest can be isolated through careful design or selection of experimental conditions.

### What do I need to consider when creating or selecting experimental conditions?

Another key question a hyperscanning researcher will face is which conditions to include in their experiment design. Much has been written about the value of clever experimental manipulations (e.g. Sternberg, 2001, 2011; Rosenbaum, 2005) and advice on how to choose among and optimize experiment designs that can be found in various sources (e.g. Ruff and Huettel, 2014). These ideas likewise apply to hyperscanning, where decisions about which conditions one includes significantly constrain data interpretation.

One particularly important consideration for joint action researchers is that one can carefully design experimental conditions to mitigate the concerns raised in Section ‘What concerns have been raised about hyperscanning?’, regarding whether inter-brain coupling effects are driven by exogenous sources (common sensory inputs) or by endogenous sources (higher-level processes). Specifically, researchers can implement experimental designs that hold one potential source of inter-brain coupling constant while systematically varying the other. Independent manipulations of participants’ reliance on exogenous and endogenous sources can be implemented by varying the experimental stimuli (or stimulation; see Section ‘How do direct manipulations of brain and behavior complement hyperscanning?’), the experimental protocol (e.g. task instructions or demands) or both. For example, some studies create ‘categorical’ manipulations of exogeneous sources by giving participants either complete access or no access to perceptual information about their partners’ actions (e.g. auditory information: Konvalinka *et al*., 2010; visual information: Tognoli *et al*., 2007). Alternatively, experimental manipulations can bias participants’ reliance on exogenous or endogenous sources to varying degrees. For example, a ‘graded’ manipulation of participants’ ability to rely on exogenous sources was implemented by Gugnowska *et al*. (2022), who manipulated performers’ familiarity with their partner’s part of a musical duet to bias people toward relying on exogenous sources (i.e. their partner’s playing, when they were unfamiliar with their partner’s part) or endogenous sources (i.e. internal memory representations, when they were already familiar with their partner’s part). Researchers should also consider including control conditions in which partners perform identical actions individually when possible. Such control conditions allow for establishing baseline levels of inter-brain coupling between partners performing the same actions without an intention to coordinate, i.e. inter-brain coupling driven by common sensorimotor inputs.

Another option for joint action researchers is to use experimental designs that investigate more open-ended interactions, such as interactions among team members (Dodel *et al*., 2011), co-pilots during different phases of flight (Astolfi *et al*., 2011) or players in cooperative card games (Astolfi *et al*., 2010). Such designs can be useful for measuring how inter-brain coupling develops over time as people converge or align interactively, or for looking at differences between experts and novices across complex tasks. Given the length of such interactions, as well as the potential for non-trial-based analyses (e.g. when long interactions cannot be epoched in a natural manner), it may not always be feasible to find proper control conditions, or they may even interfere with the natural unfolding of studied interactions. In this case, one may employ a control of random estimates computed from shuffled or permuted pairs to contrast against real pairs (see Section ‘How can I define what is meant by “chance” statistical levels?’). Taken together, careful consideration of experimental design can facilitate subsequent interpretation of whether, how and why inter-brain coupling occurs during a given joint action.

### Are we there yet? What else do I need to consider when designing a hyperscanning study?

Additional practical decisions concerning basic technical aspects of data acquisition will need to be made when designing a hyperscanning study. Luck (2014; see especially Chapter 5) provides a guide for recording clean EEG signals, and Keil *et al*. (2014) provide guidelines for recording, analyzing and reporting EEG (and MEG) data. Cohen (2014; see especially Chapter 6) discusses considerations for designing EEG experiments that are appropriate for time frequency-analyses (see also Sections 4 and 5). A critical issue for hyperscanning studies is to ensure that EEG recordings are precisely synchronized across participants; Barraza *et al*. (2019) provide instructions for how to set up commercially available EEG hardware and recording software to do so. Likewise, care must also be taken to ensure that stimulus presentation is precisely synchronized if presented to participants on separate computer screens. Additional useful resources include guidelines for conducting fNIRS hyperscanning studies (e.g. Hu *et al*., 2021) and for carrying out EEG hyperscanning in developmental contexts (i.e. with infants and children; Turk *et al*., 2022).

We note here two important practical issues that need to be given special consideration for the design and interpretation of EEG hyperscanning experiments in joint action contexts. First, when measuring inter-brain synchronization using EEG methods, there may be differences in signal-to-noise ratios across compared conditions, which can greatly affect phase estimates (van Diepen and Mazaheri, 2018), and consequently phase-based inter-brain estimates, such as phase-locking values and circular correlations (see Zimmerman *et al*., 2023, for an in-depth discussion). For example, many studies have reported inter-brain synchronization across alpha and mu oscillations (Dumas *et al*., 2010; Goldstein *et al*., 2018; Chen *et al*., 2023); but occipital alpha amplitudes are suppressed when people attend to a visual stimulus (Bazanova and Vernon, 2014), just as the Rolandic mu-rhythm amplitude is suppressed when people produce movements (Gastaut and Bert, 1954). This is problematic if a study compares interaction conditions—where people move together, or jointly attend to visual stimuli, and hence exhibit high alpha or mu suppression, and as a result exhibit low signal-to-noise ratios—with conditions where people do not interact, and are hence less attentive, exhibiting higher signal-to-noise ratios across alpha-mu oscillations (Zimmermann *et al*., 2022). These signal-to-noise differences between conditions have been shown to drive differences in inter-brain amplitude- and phase-based estimates between conditions (Zimmerman *et al*., 2023). Practically, this concern can be mitigated by carefully selecting control conditions that are as similar as possible to the experimental conditions of interest and allow appropriate baseline measurements against which to compare the latter (see also Section ‘What do I need to consider when creating or selecting experimental conditions?’). Further guidelines for assessing and optimizing signal-to-noise ratios when analyzing neural oscillation data are provided by Donoghue *et al*. (2022).

A second important practical consideration to be made when designing hyperscanning studies is the potential for noise coupling between the EEG recording equipment and stimulus sources and/or sensors (e.g. response buttons). When sensors from several participants are connected to the same sensor hub (i.e. computer), there is a risk of transferring noise between participants through the sensor system. Such coupling can cause temporally aligned noise in the participants’ EEG recordings, which could be misinterpreted as inter-brain synchronization. Considerations about noise coupling are especially important when using dry-contact EEG electrodes, which are more susceptible to noise interference than traditional wet electrodes (Kappel *et al*., 2019).

To mitigate noise coupling concerns, wiring must be done carefully; wires relating to sensors and/or stimuli must be spatially separated from wires related to the EEG recording equipment, to reduce capacitive and inductive coupling between the wires (Webster and Nimunkar, 2020). Furthermore, in some cases it might be necessary to galvanically isolate each sensor from the sensor hub (i.e. ensure that no electrical current flows between them) using a digital isolator. Commercial isolators are available for most communication protocols (e.g. USB); however, in special cases, it might be necessary to implement galvanic isolation with custom-made hardware. Finally, testing noise coupling in an experimental setup can be challenging, and methods will vary depending on the sensor and stimulus types. Potential methods that can be employed include (i) performing electrical tests of the equipment, e.g. to ensure that no current is flowing between two electrical domains that are galvanically isolated, and/or (ii) including control conditions in which sensors are activated and stimuli are presented but the neural response of interest is not expected.

Once a researcher has designed their experiment and collected their data, the next questions they face concern how best to analyze that data. We turn to these considerations next.

## Hyperscanning projects can yield intimidating datasets. How do I hone in on the dependent variable(s) that are best suited to answer my research question?

Hyperscanning projects yield rich data, and it can be difficult to know which dependent variable(s) to choose among the many different options available. When conducting confirmatory studies, making these decisions in advance and pre-registering them when possible can help guard against concerns about *p*-hacking and hypothesizing after the results are known (HARKing; see Section ‘What issues will I need to consider as I progress through the stages of analyzing my hyperscanning data?’ for further discussion and see Holroyd, 2022, for discussion of these concerns with respect to hyperscanning in particular). In Section ‘How do I choose among the many different options for inter-brain measures?’, we discuss considerations for choosing among different inter-brain measures. In Section ‘How do I select which frequency bands, spatial locations, and time windows to analyze?’, we discuss considerations for making decisions about which frequency band(s), spatial locations and time windows to analyze. We discuss alternative data-driven approaches for data analysis in Section ‘What issues will I need to consider as I progress through the stages of analyzing my hyperscanning data?’.

### How do I choose among the many different options for inter-brain measures?

Choosing which inter-brain measures to use can be a significant challenge because inter-brain analyses are still in their infancy, and therefore there is not yet a clear standard in the field for which measures to use in different circumstances. Czeszumski *et al*. (2020) and Hakim *et al*. (2023) provide overviews of state-of-the-art measures for comparing activity between brains and describe the technical details of these analyses. Ayrolles *et al*. (2021) have implemented most of these measures in an opensource Python-based inter-brain analysis toolbox, HyPyP, allowing easy accessibility even to researchers without the technical knowledge required to program these analyses from scratch. Ayrolles *et al*. (2021) categorize inter-brain measures into five broad categories (‘Phase synchrony’; ‘Amplitude/envelope correlation’; ‘Coherency-based’; ‘Causality measures’; ‘Other’) and provide a comprehensive summary for researchers. Although many of these inter-brain measures are adapted from standard measures of functional connectivity in the single-subject EEG/MEG literature (for an overview see Hipp *et al*., 2012), for joint action researchers new to hyperscanning—particularly those without backgrounds in neurophysiology, physics or engineering—it might not be obvious how to choose among them. Here, we briefly summarize each measure and provide advice about when it is appropriate to use each measure and why one might select a given measure over the other options available.

#### Why choose phase synchrony?

##### What is phase synchrony?

**Fig. 2.**
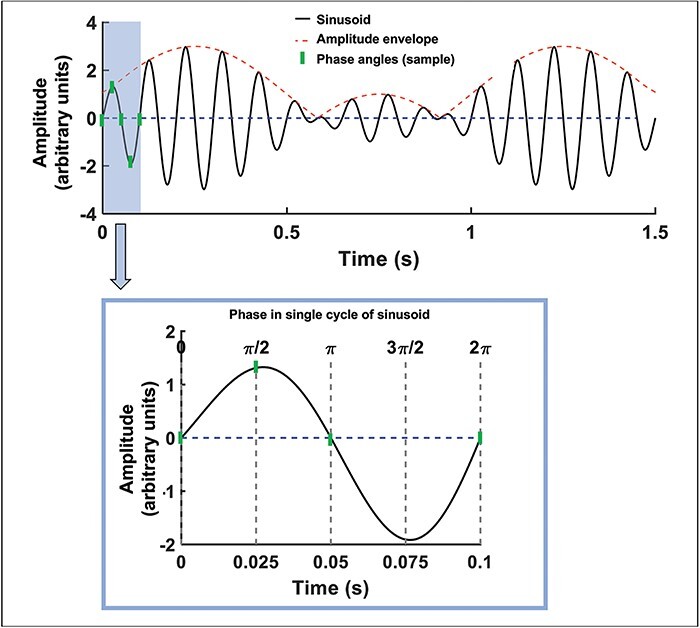
Sinusoidal signal illustrating key measurement concepts. ‘Phase’ corresponds to the position of the oscillation at a particular point in its cycle, from 0 to 2π. ‘Frequency’ corresponds to the speed of the oscillation, measured in Hz (cycles per second; here the oscillation frequency = 10 Hz). ‘Amplitude’ corresponds to the maximum value of the oscillation relative to a reference (in this example, the reference is zero). The ‘amplitude envelope’ is a function that captures fluctuations in the oscillation’s amplitude over time, commonly computed using the ‘Hilbert transform’.

**Fig. 3.**
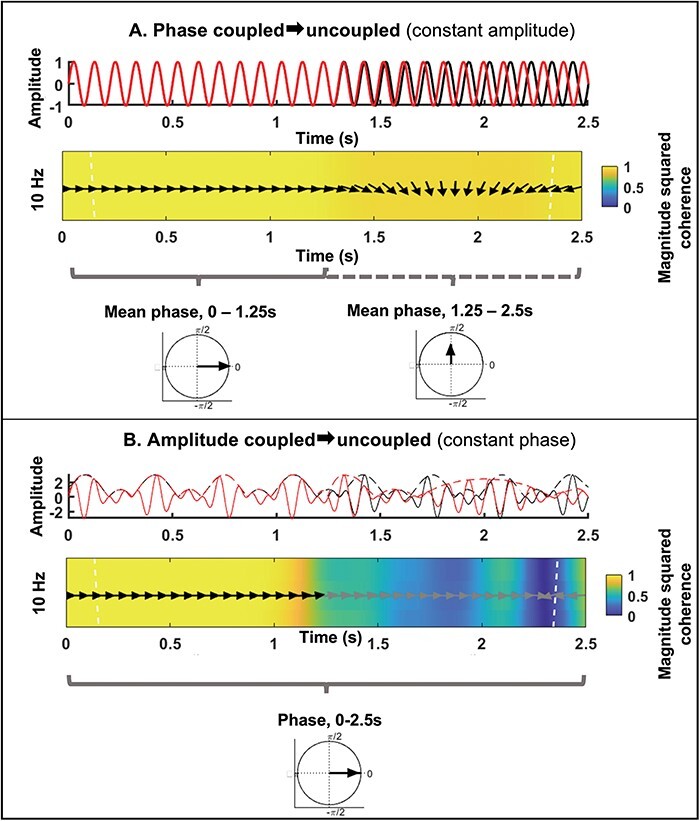
Illustration of phase and amplitude coupling between two 10 Hz signals. Panel A (top): Two 10 Hz sinusoidal signals of 2.5 s duration that are phase-locked (0-degree offset) from 0 to 1.25 s. From 1.25 to 2.5 s, one of the signals (red) displays linear phase drift until the phase offset between the two signals is approximately equivalent to *pi*. Panel A (middle): Wavelet coherence plot displaying phase drift (arrows) and coherence (color scale) between the two signals shown in Panel A, top. Rightward arrows aligned with the horizontal axis indicate an offset of 0 degrees, and leftward arrows aligned with the horizontal axis indicate an offset of *pi*. Dashed white lines indicate the boundary of the Cone of Influence, where the wavelet analyses are less accurate due to edge artifacts (phase and coherence values should not be interpreted outside the Cone of Influence). Panel A (bottom): Unit circles depicting the mean phase offset (black line, called the ‘resultant vector’) between the two signals from 0 to 1.25 s (left) and from 1.25 to 2.5 s (right). Phase consistency is indicated by the length of the resultant vector, also referred to as ‘mean vector length’. Panel B (top): Two 10 Hz signals (fully phase-locked, 0-degree offset) that are amplitude coupled from 0 to 1.25 s. Both signals are 10 Hz oscillations that are amplitude modulated by a 3 Hz modulation signal. Solid lines denote the signal and dashed lines denote the amplitude envelope. From 1.25 to 2.5 s, the amplitude modulation frequency of one of the signals (red) drifts continuously, causing the amplitudes of the two signals to become uncoupled. Panel B (bottom): Wavelet coherence plot of the two signals shown in Panel B, top. As in Panel A, middle, arrows represent phase offsets, color represents coherence and dashed white lines indicate the boundary of the Cone of Influence. Amplitude uncoupling of the two signals is clear from the change in coherence at 1.25 s, and arrows change color to denote change in coherence (phase between incoherent signals may not be informative).

Phase is the position of an oscillation in its cycle at any given point in time, as illustrated in Figure 2. Two or more oscillations are considered to display phase synchrony if the offset of their instantaneous phase is constant over some time window of interest (Lachaux et al., 1999), as illustrated in the top panel of Figure 3A. Inter-brain phase synchrony is therefore defined as inter-brain alignment of instantaneous phases of neural oscillations measured from two or more people. A common approach to measuring phase synchrony is to extract the instantaneous phase of the waveform in question at each point in a time-window of interest (e.g.Bruns and Eckhorn, 2004), and then compute the Phase Locking Value (PLV; Lachaux et al., 1999), the Phase Lag Index (PLI; Stam et al., 2007) or the Circular Correlation Coefficient (CCorr; Jammalamadaka and Sengupta, 2001; Burgess, 2013) between the two waveforms’ instantaneous phases (also referred to as phase coherence; Mormann et al., 2000). Details about how these measures are typically calculated, and further discussion of their use in hyperscanning paradigms, are provided in the Supplementary Material S1.

##### When would I use phase synchrony measures?

Phase synchrony is a particularly suitable choice for joint action paradigms in which the temporal relationship between partners’ actions is crucial to the task (e.g. duet music performance, in which partners’ actions must be aligned in time to produce synchronous tone onsets, or a joint reaching task, in which partners must simultaneously move towards for a target), and the behavioral dependent variable is therefore the temporal offset between partners’ actions. In these cases, the researcher might reasonably hypothesize that the temporal offset between partners’ actions will be reflected in corresponding offsets in cortical activity surrounding the joint action. Cortical phase synchrony—measured as the PLV, PLI or CCorr (see Supplementary Material S1)—would thus be reasonable to measure before, during and/or after the joint action, in a frequency band hypothesized to be relevant to the specific behavior (see Section ‘How do I select which frequency band(s) to analyze?’).

Phase synchrony is better suited than other inter-brain measures when the researchers’ primary interest is in fine-grained temporal relationships between partners’ brain activity in a joint action task. Phase changes can occur at the millisecond timescale, and therefore can provide high-precision information about inter-brain temporal dynamics. However, phase measurements are thus also highly susceptible to temporal jitter in measurements (e.g. latency variability that naturally arises in increasingly popular setups where measurements are synchronized via software rather than hardware). Thus, if researchers are using equipment for which the measurement jitter exceeds expected behavioral phase differences (e.g. tone onset asynchronies in music performance), then options include either only cautiously interpreting phase-locking measures, and/or instead selecting an inter-brain measure that is less susceptible to measurement noise but still a logically relevant neural correlate of the behavior of interest (e.g. amplitude or power coupling, discussed next).

#### Envelope coupling

##### What is envelope coupling?

As illustrated in Figure 2 and the top panel of Figure 3B, the envelope of a waveform provides an estimate of how an oscillatory signal changes in amplitude or power (power ∼= amplitude squared) over time. An oscillation that does not change amplitude over time would have a flat envelope, whereas an oscillation that fluctuates rhythmically in amplitude every 1s would display an oscillatory envelope with a 1 Hz rate of change (Hz = cycles per second). When two signals exhibit envelope coupling, it means that their envelopes show corresponding patterns of change in amplitude over time (Bruns and Eckhorn, 2004). For example, as illustrated in the first half of the timeseries shown in Figure 3B, when the envelopes of two oscillations change amplitude at the same time, then these signals display envelope coupling. Conversely, if the envelopes of two oscillations change at random intervals relative to one another, then these signals do not display envelope coupling, as illustrated in the second half of the timeseries shown in Figure 3B. Critically, envelope coupling is not dependent on the absolute amplitude of the envelopes (i.e. the amplitude of envelopes does not have to be on the same scale); it is only dependent on whether the two envelopes display corresponding time-courses of amplitude change. Details regarding how envelope coupling is measured can be found in the Supplementary Material S2.

##### When would I use envelope coupling?

Envelope coupling would be well applied to joint action paradigms concerned with measuring correlated changes in the frequencies of partners’ behavior (e.g. dyadic dance in which partners must change their tempo of motion, or joint music performance in which partners’ movements change frequency as they produce notes of different durations). In these cases, it would be expected that the envelopes of cortical oscillations would track changes in the frequencies of partners’ behavior. For example, if musical partners alternately produced tone onsets at 2 Hz and 4 Hz, representing the beat frequency and eighth-note subdivisions, respectively, then it would be expected that both partners’ envelopes should track these frequency changes and therefore be coupled in the 2 Hz and 4 Hz frequency ranges, respectively.

Envelope coupling could also be used to measure coupling between each participant’s intra-brain activity and external stimuli (e.g. as a baseline measure or in certain control conditions), similar to its use as a measure of attention to an auditory source in listening studies (e.g. Mirkovic *et al*., 2016). Envelope coupling can also be used to calculate inter-subject correlations (ISCs), which are often used in studies of stimulus-driven coupling between subjects who view the same stimulus separately (e.g. Dmochowski *et al*., 2012).

Envelope coupling can occur even when two oscillatory signals do not display strong phase synchrony (Bruns *et al*., 2000), and is therefore well-suited in cases where phase relationships might not be possible to measure precisely (e.g. due to signal-to-noise concerns or temporal jitter; see Section ‘When would I use phase synchrony measures?’). Some work indicates that envelope correlations have higher reproducibility than other measures of coupling (Colclough *et al*., 2016), perhaps due to the robustness of amplitude/power measures to measurement concerns.

Notably, envelope coupling measures are not ideal for paradigms where the envelope of a cortical oscillation is not expected to change over time (e.g. when perceiving a perfectly periodic stimulus rhythm such as an isochronous series of tones where there is no frequency change), because in these cases, the envelopes associated with that oscillation are likely to remain flat and therefore do not reflect dynamic changes that might be coupled between brains. Taken together, envelope coupling is best applied when researchers are interested in how cortical activity reflects changes in the frequency of partners’ behaviors over time, and/or when precise phase relationships of behavioral and/or brain signals may not be possible to measure.

#### Wavelet coherence measures

##### What is wavelet coherence?

Wavelet coherence measures are illustrated in the middle panels of Figure 3A and B. As these panels show, two brain signals can fluctuate between being highly coupled with regard to both phase and amplitude in a specific frequency band (as illustrated in the first half of the timeseries in each panel) and being decoupled along one or both dimensions (phase and/or amplitude, as illustrated in the second half of the timeseries in each panel). Coupling of phase and/or amplitude can occur in one or more frequency bands while being uncoupled in others (this is not illustrated in Figure 3, which shows only a single narrow frequency band around 10 Hz. See Rösch and Schmidbauer (2016) for examples and advice for interpreting wavelet coherence plots across multiple frequency bands). Cross-wavelet coherence analysis allows for assessing frequency-band-specific phase- and/or amplitude-coupling by providing measures of phase and amplitude similarity across the frequency spectrum (Grinsted et al., 2004). Thus, wavelet coherence can be computed between brains at a single EEG electrode site, for example, to assess whether partners in a hyperscanning study display local correlations of amplitude and/or phase. Supplementary Material S3 briefly introduces how wavelet coherence is measured.

##### When would I use wavelet coherence?

Wavelet coherence is typically used in cases where the researcher is interested in whether partners’ brains display correlated changes in phase and/or frequency over some time window of interest. For example, if researchers are interested in whether EEG beta and alpha power increase in magnitude several hundred milliseconds after a stimulus event such as a musical tone, then wavelet coherence analyses would be well applied to assess the temporal dynamics of oscillatory power in these multiple frequency bands in the time window of interest.

Wavelet coherence is preferable to other measures when researchers are (i) interested in measuring phase synchrony/coupling across a wide number of frequency bands but want a more parsimonious method than narrow-band filtering signals at multiple frequencies and then computing phase synchrony and/or envelope coupling, and/or (ii) when researchers are interested in simultaneously investigating frequency and phase dynamics because they have *a priori* hypotheses about both measures and how they relate to behavior.

#### Directional measures

##### What are directional measures?

While phase synchrony, envelope coupling and wavelet coherence are symmetrical measures, directional (or causality) measures such as Granger Causality, Partial Directed Coherence (PDC) and transfer entropy measure direction of information flow between paired individuals—person A and person B—and hence yield different inter-brain coupling estimates from A to B than from B to A (Ayrolles et al., 2021). Granger Causality is a well-established method for quantifying whether one time-series can predict another time-series, or as per its definition, ‘an observed time series x_j_(n) Granger-causes another series x_i_(n), if knowledge of x_j_(n)’s past significantly improves prediction of x_i_(n)’ (Granger, 1969). Importantly, the relationship between x_i_(n) and x_j_(n) need not be reciprocal, such that if x_i_(n) Granger causes x_j_(n), this does not imply that x_j_(n) Granger causes x_i_(n). For inter-brain analyses, the most common directional measure used is PDC, which is a Granger Causality measure in the frequency domain (Baccalá and Sameshima, 2001). PDC measures the degree of influence that activity measured in a region of interest (ROI) from one partner has on a ROI measured from another partner across different frequencies of interest, and has been widely employed across EEG hyperscanning studies (e.g.Astolfi et al., 2010; Babiloni et al., 2006; Leong et al., 2017). Further details on the measurement of PDC can be found in Supplementary Material S4.

##### When would I use directional measures such as PDC?

Directional measures such as PDC are relevant when a directional influence from one person to another is expected, resulting in an asymmetric flow of information. For example, if there is a leader***–***follower relationship such that person A is leading (or initiating) an interaction, while person B is following, one might expect there to be directional coupling of neural oscillations, such that person A’s EEG activity (in some ROI) ‘Granger-causes’ person B’s EEG activity. This type of influence has previously been demonstrated in a study that employed a collaborative card game paradigm, which resulted in directional coupling in the beta frequency range from EEG activity in the anterior cingulate cortex (ACC) of the leader to EEG activity in the right prefrontal and parietal areas of the leader’s partner (F. Babiloni et al., 2006).

Non-directional measures yield reciprocal coupling (with no difference from A to B and B to A), and hence cannot capture asymmetric relationships between brain activity across interacting partners. Directional measures would thus be useful for capturing leader–follower or other asymmetric relationships on a neural level and would be selected in cases where such an asymmetric relationship exists between interacting partners on a behavioral level.

In addition to choosing among different measures as presented in Section ‘How do I choose among the many different options for inter-brain measures?’, researchers must also select which frequency bands, spatial locations and time windows to include in their analyses. We provide considerations for making decisions about these aspects of the dependent variable next.

### How do I select which frequency bands, spatial locations and time windows to analyze?

We begin this section by noting that considerations regarding which frequency bands, spatial locations and time windows to analyze apply to both inter-brain and intra-brain data. We recommend that researchers consult recommendations for frequency and time-frequency analyses of intra-brain data provided by Keil *et al*. (2022) and Donoghue *et al*. (2022), as many of these recommendations also apply in analyses of inter-brain data. We note selected recommendations below, while focusing our discussion primarily on issues relevant to hyperscanning studies of joint action.

#### How do I select which frequency band(s) to analyze?

Joint action requires the alignment of sensorimotor behavior between partners and can also involve a range of higher-level cognitive processes such as planning, error-monitoring, joint attention and perspective-taking (Keller et al., 2014; Bolt and Loehr, 2021; Sebanz and Knoblich, 2021). Accordingly, researchers will want to select EEG frequency bands that capture the sensorimotor and/or cognitive processes that are the focus of their research questions.

The selection of frequency bands associated with sensorimotor processes can be based on the wide literature addressing EEG correlates of solo action observation, imagery, planning and execution (for a review, see Salenius and Hari, 2003); the literature has identified key cortical oscillations, namely, Rolandic mu (which has two components, one centered ∼10 Hz and the other at ∼20 Hz; Hari, 2006) and beta (13–35 Hz; Jensen *et al*., 2005) oscillations, that both display stereotypical spatiotemporal patterns time-locked to actions. Specifically, oscillatory power decreases over contralateral sensorimotor brain areas prior to actions (termed event-related desynchronization, ERD) and then increases relative to baseline levels over bilateral motor-related areas after actions (event-related synchronization, ERS; for a review see Pfurtscheller and Da Silva, 1999), and to a lesser degree over ipsilateral sensorimotor brain areas (Nikouline *et al*., 2000). ERD and ERS during action observation have been proposed to reflect a common coding of perception and action, as well as motor prediction processes (Arnal and Giraud, 2012) that may underlie joint action coordination (Kourtis *et al*., 2013).

A growing body of hyperscanning research has explored the role of sensorimotor oscillations in joint action coordination and planning (for a review, see Bolt and Loehr, 2021). Furthermore, as already noted in Section ‘How do direct manipulations of brain and behavior complement hyperscanning?’, Novembre *et al*. (2017) provide compelling evidence that cortical motor-related beta oscillations may play a ‘causal’ role in joint action coordination. Thus, mu and beta frequencies are excellent targets for answering questions related to sensorimotor planning and coordination in EEG hyperscanning paradigms.

In contrast with sensorimotor processes, higher-level cognitive processes underlying joint action are highly heterogeneous and frequency band selection can therefore be a major challenge for researchers who are interested in higher-level mechanisms of coordination. It is therefore a good practice to select frequency bands based on literature describing how cortical oscillations contribute to higher-level processes in individual behavior (for overviews, see Başar *et al*., 2001; Ward, 2003; Hari and Puce, 2017).

A more data-driven approach to identifying frequency bands of interest—specifically in joint action tasks involving rhythmic behaviors such as music performance—is to implement ‘frequency-tagging’ of partners’ EEG data. Frequency-tagging quantifies the magnitude of steady-state evoked responses elicited in a given task (Nozaradan *et al*., 2011). Varlet *et al*. (2020) used this approach in a visuo-motor interpersonal coordination task to identify brain activity related to unique frequencies of flickering LEDs attached to each individual (‘Self/Other’ frequencies) and to identify brain activity associated with the combined frequency (the sum of their individual frequencies), called the ‘inter-modulation frequency’, as an index of self-other integration. If brain activity arises at Self/Other/Intermodulation frequencies during joint action, then frequency-tagging should reveal clear spectral peaks in partners’ EEG data at these frequencies, providing empirical support to focus EEG analyses on oscillations at these frequencies.

Finally, one important general consideration when selecting frequency bands is that the peak frequency of a given oscillation can vary between participants and within participants across different tasks (Donoghue *et al*., 2022; Keil *et al*., 2022). For this reason, analyses based solely on canonical frequency ranges (e.g. 8–12 Hz for alpha or 13–30 Hz for beta) may lead to misestimations. Donoghue *et al*. (2022) provide guidelines for checking how well frequency bands that are selected *a priori* match actual peaks in the power spectra in a given dataset, and for how to calculate individualized frequency bands if needed.

#### How do I select which spatial location(s) to analyze?

Another key question is which electrode sites or underlying source regions should be selected to measure the sensorimotor and/or cognitive processes of interest. Ignoring this question can lead to problematic multiple comparison issues (see Luck and Gaspelin, 2017). One option is to define ROIs*a priori* based on prior literature. When research questions are related to sensorimotor, perceptual or cognitive processes that have been previously studied in individual behavior, it may be relatively straightforward to select appropriate ROIs. For example, as mentioned in Section ‘How do I select which frequency band(s) to analyze?’, sensorimotor mu and beta activity are typically observed most prominently over central electrodes contralateral to the side of the body used for motion (Pfurtscheller and Da Silva, 1999).

However, it can be challenging for joint action researchers to define ROIs based on prior literature when addressing novel questions and/or using non-standard paradigms that may be simultaneously influenced by multiple sensorimotor and cognitive processes. One option in such cases is to measure EEG activity in ‘localizer’ tasks that target the constituent processes that comprise the joint task of interest. One can then identify how oscillations of interest are spatially distributed in each of these localizer tasks to define subsequent analysis regions. For example, if a researcher is interested in studying neural correlates of joint improvisation during dance, they could run localizer tasks in which participants perform solo dance with and without music, observe a partner dancing with and without music, etc. Each task would reveal how a given oscillation of interest is distributed across electrodes when participants perform one constituent feature of joint dance improvisation. These spatial patterns could then be used to create ROIs or spatial filters (see Blankertz *et al*., 2007, for further information about spatial filtering) that can be applied when analyzing data from the joint task.

A general consideration here is that researchers will need to decide whether to define ROIs in sensor space (i.e. in terms of EEG signals recorded at specific scalp electrodes) or source space (i.e. model-based estimates of the probable neural generators of these signals). Donoghue *et al*. (2022) provide guidance regarding when and how to complement analyses in sensor space with analyses in source space, and Keil *et al*. (2022) provide advice regarding how to ensure interpretable results when combining source estimation with frequency or time-frequency analyses.

#### How do I select time window(s) for analysis?

Finally, researchers will also need to decide how long their analysis time windows (also referred to as epochs) need to be to capture the brain dynamics of interest. This will depend on factors that include the joint action being investigated and the frequency bands selected. Here, we suggest several key questions researchers can consider to help guide their decision-making.

##### Question 1: How many cycles of the oscillation of interest would be required to detect a meaningful pattern of correspondence between partners’ brain activity?

The necessary time windows can be shorter at higher frequencies (which by definition entail more cycles per second) than at lower frequencies (which entail fewer cycles per second). Keil *et al*. (2022) and Keil *et al*. (2014) provide further discussion of this and other basic issues (e.g. trade-offs between time and frequency resolution, edge artifacts, etc.). Additional considerations include that certain measures require a sufficient number of cycles to detect the pattern of interest (e.g. correlation-based measures such as amplitude envelope correlations require enough cycles to detect a correlation). Furthermore, if the selected time window only captures a few cycles of oscillation, there is a risk that partners’ brain activities will appear to be more synchronized by chance, given that they oscillate at similar frequencies, and are hence more prone to spurious coupling (Zimmerman *et al*., 2023).

##### Question 2: What is the rate at which the dependent variable is expected to change?

Many inter-brain measures capture features of oscillations that change at a slower rate than the oscillation itself. For example, amplitude envelopes of oscillations typically change at a slower rate than oscillations themselves. Therefore, if researchers want to investigate inter-brain amplitude coupling, they should consider the rate at which amplitude changes for a given oscillation. The rate at which a given dependent variable changes is often linked to the behavior in question, as discussed next.

##### Question 3: What is a behaviorally meaningful time interval?

The answer to this question is relevant to Question 2 because changes in dependent variables are often time-locked to changes in behavior. Furthermore, many studies divide continuous data into evenly spaced epochs without a clear justification for the size or spacing of the epochs. We suggest that selecting behaviorally meaningful time windows could be a more useful strategy when conducting theoretically motived analyses. For example, when investigating music performance, behaviorally meaningful time windows could correspond to musical bars, phrases or entire melodies. Another option is to identify repeating segments of behavior in the joint action of interest (e.g. musical bars or phrases) and use the mean length of these segments to define the time window length. This can help ensure that when inter-brain measures are averaged across epochs, they capture neural activity from comparable segments of behavior. This will, in turn, improve signal quality.

##### Question 4: Are there any stationarity considerations?

When longer time windows are selected for analysis, the stationarity (stability over time) of the oscillatory activity can be an issue. Donoghue *et al*. (2022) provide an overview of implications and recommendations for addressing stationarity in spectral analyses, and Ayrolles *et al*. (2021) summarize the stationarity requirements for the most common inter-brain measurements. Here, we suggest that researchers can consider whether the behavior being measured (see Question 3) might impact stationarity, and/or explicitly include investigating how patterns of inter- or intra-brain activity change dynamically over the course of a joint action among their research questions.

## What issues will I need to consider as I progress through the stages of analyzing my hyperscanning data?

In this final section of the hyperscanning guide, we discuss considerations that are faced during data analysis, including when pre-processing the data, inspecting the data, performing statistical analyses and visualizing their results. It is important to consider these issues, particularly those related to what kinds of statistical analyses the study design will permit or require, during the design phase of a hyperscanning project. Doing so can help researchers avoid discovering confounds in the design only after the data have been collected. Researchers can also consider pre-registering their data analysis plans when possible. Such explicit advance-planning mitigates against practices that have contributed to the so-called replication crisis plaguing the fields of psychology and cognitive neuroscience (Pashler and Wagenmakers, 2012; Kelly and Hoptman, 2022). These practices include HARKing, where post-hoc interpretations of chance findings (false positives) are allowed to masquerade as *a priori* theories (Kerr, 1998), and unfettered multiple comparisons (i.e. exhaustively comparing all experimental conditions without theoretical motivation), which increases opportunities for false positives (Luck and Gaspelin, 2017; Midway *et al*., 2020).

### Where can I find recommendations for pre-processing my EEG data?

Numerous pre-processing steps need to be applied to EEG data, including filtering, re-referencing, checking for and correcting or rejecting artifacts, selecting appropriate baselining procedures and potentially performing spatial transformations (e.g. source estimation). Recommendations for carrying out these pre-processing steps are provided elsewhere (e.g. Cohen, 2014; Luck, 2014; Keil *et al*., 2022), but are dependent on the type of analysis, so we advise focusing on recommendations that are specific to the analyses one plans to conduct (e.g. time-frequency *vs* ERP analyses).

### What should I consider when initially inspecting the data?

Prior to carrying out analyses of intra- or inter-brain data, joint action researchers will likely want to examine behavioral measures to determine whether expected patterns of behavior are present in their experimental tasks and conditions. For example, it might need to be established that the experimental manipulations yielded the expected patterns of behavioral differences between conditions.

Next, prior to carrying out statistical analyses of intra-brain data or calculating inter-brain measures between partners, researchers will need to check the intra-brain data to determine whether the neural oscillations of interest are actually present. Donoghue *et al*. (2022) discuss numerous issues that can arise when examining intra-brain neural oscillations and recommend steps to address them. We summarize two intra-brain data checks that are crucial to carry out before analyzing inter-brain data below.

#### Intra-brain data check 1: Does each partner display a spectral peak in the target frequency band?

One critical step in assessing the intra-brain data is to establish that each partner displays oscillations at the frequency of interest, at the selected analysis location (e.g. electrode cluster). Researchers often assume that oscillations of interest are present, and it is important to acknowledge that this assumption might not be true. This assumption can be checked by computing a spectral analysis of each partner’s EEG data and plotting the results at the analysis site of interest. Ideally, a spectral peak should be observed in the frequency band of interest in each partner’s spectrum. We provide an illustration of this strategy in our discussion of data visualization in Section ‘What visualizations should I include when I am ready to present my results?’. One can also apply mathematical or statistical techniques to confirm the presence of oscillatory activity (see e.g.Nozaradan *et al*., 2011; Donoghue *et al*., 2022).

#### Intra-brain data check 2: Does the associated scalp topography reflect a stereotypical spatial pattern?

Demonstrating a spectral peak at the frequency of interest is not sufficient to conclude that a particular participant displays the oscillation of interest. For example, peaks in the alpha band can reflect different underlying processes arising from different brain areas, e.g. sensorimotor *vs* attention-related alpha activity. Moreover, peaks in the beta or higher frequency bands can often reflect local artifacts rather than true brain processes. Plotting each participant’s scalp distribution of amplitude/power at a target frequency can provide insight into whether the oscillation captured in the spectrum reflects the brain process of interest (as also illustrated in Section ‘What visualizations should I include when I am ready to present my results?’). For example, if a researcher is interested in sensorimotor beta oscillations, then the scalp topography underlying peak beta activity for each participant should reflect maximal activity at frontocentral (usually lateralized) sites. If a plot of the scalp distribution of a participant’s peak beta activity shows maximal (and focal) power over electrodes near the jawline (e.g. TP9/10), then this participant’s beta peak likely reflects mainly noise and should not be considered for analysis.

If a participant’s intra-brain data do not pass both of the above checks, then it is advisable to exclude the participant (and their partner) from inter-brain analyses, as one cannot conclude that what is being measured is the oscillatory signal of interest. If many participants do not pass these intra-brain checks, then it is advisable to revisit the design and/or measurement methodology, as any inter-brain coupling that might be detected in such data is likely to be spurious.

### What issues do I need to consider when selecting statistical analysis strategies for hyperscanning data?

Several resources provide overviews of statistical analysis issues and recommendations for intra-brain analyses (e.g. Maris, 2012; Keil *et al*., 2014; Luck and Gaspelin, 2017). For example, Keil *et al*. (2022) provide an overview of statistical approaches that are often applied when analyzing intra-brain frequency and time-frequency data, including univariate, mass univariate, Bayesian and machine learning approaches. Here, we focus on several questions that researchers might face when planning and carrying out statistical analyses for a hyperscanning study.

#### Do my statistical models capture the multi-level structure of joint action data?

One issue that must be considered when planning statistical analyses for hyperscanning studies is how to account for the multi-level structure of the data, whereby individual participants are nested within pairs or within groups. This issue is relevant not just for hyperscanning studies, but for any joint action study that assesses individual measures from members of pairs or groups. The key issue here is that data from individual group members are not independent (e.g. due to shared stimulus effects, affiliation effects, etc.). Therefore, multi-level mixed-effects models that include pair or group as a random factor within which individual participants are nested are likely more appropriate than Analysis of Variance, which assume that cases are independent. There are a number of useful resources researchers can consult regarding why and how to use multi-level mixed-effects models (e.g. among others:West et al., 2006; Kenny et al., 2020; Nezlek and Mroziński, 2020; DeBruine and Barr, 2021).

#### How can I define what is meant by ‘chance’ statistical levels?

As noted in Section ‘What do I need to consider when creating or selecting experimental conditions?’, researchers sometimes use shuffled or permuted pairs as a control against which to assess inter-brain coupling in naturalistic or open-ended joint actions. Essentially, in this strategy, participants’ data are shuffled or permuted to establish ‘chance’ levels against which to compare the level of inter-brain coupling observed in real pairs. Example studies that have used this strategy include A. Zamm et al. (2021), Gugnowska et al. (2022) and Reindl et al. (2022). A critical consideration when using this strategy is that defining what is meant by ‘chance’ is challenging for inter-brain analyses. As discussed in Section ‘What concerns have been raised about hyperscanning?’, inter-brain coupling can arise from exogenous sources (e.g.both members of a pair receiving the same sensory input) and endogenous sources (e.g. higher-level cognitive or social processes). The definition of chance depends on whether one is interested in exogenous or purely endogenous coupling. For exogenous coupling, chance can be defined as what one would expect between two participants exposed to different stimuli. In contrast, when researchers are interested in endogenous coupling, they typically treat exogenous coupling as chance, by comparing inter-brain coupling between partners who performed a task together with coupling between ‘surrogate’ partners (who performed the same task but separately, such that any observable inter-brain coupling can only be due to exogenous influences). Notably, the choice of chance estimate is a way that researchers can establish whether they have indeed found evidence of endogenous coupling in a hyperscanning study.

Although these chance definitions may seem straightforward in theory, stumbling blocks can arise in practice. A common issue is that in joint action studies the ‘stimulus’ is often produced by the participants themselves, e.g. all pairs in a study will co-produce the same melodic stimulus, such that the stimulus timing is not identical between pairs. Therefore, inter-brain coupling may be higher between true partners (who completed the task together) relative to surrogate partners (who completed the task separately), solely due to slight differences in how the stimulus is produced between pairs. Therefore, exogenous inter-brain coupling can be statistically mistaken for endogenous coupling due to slight variations in how pairs produce nominally (but not actually) identical stimuli. There is often not a clear answer to this problem, but it is crucial to consider it when designing the study and planning the analyses, as appropriate control conditions can often help provide good work-around solutions.

#### What if I want to employ a data-driven approach to analysis?

Some research questions or experimental designs call for a more data-driven approach to analysis. For example, some studies seek to predict which neural features (at the intra- or inter-brain level) are best predictors of successful interaction or of particular joint action effects. Machine learning (ML) approaches can be useful for extracting such patterns and may be particularly beneficial in the absence of specific *a priori* neural hypotheses. ML methods have previously been successfully used to decode brain states of individual brains (Haynes and Rees, 2006), but have also been proposed as a beneficial way to address the two-brain challenge (Konvalinka and Roepstorff, 2012)—namely, to address whether more information can be gained about neural mechanisms by looking at two brains in interaction rather than the sum of individual brains. For example, multivariate decoding has been successfully used to classify mutually *vs* unidirectionally interactive conditions from two-brain EEG data in interacting pairs (Konvalinka et al., 2014), based on EEG features that were able to distinguish leaders and followers that emerged from the interaction. This ML approach was useful in picking up differences in brain activity in either member of each interacting pair, essentially distinguishing leaders from followers during interaction based solely on 10 Hz power. This was informative in elucidating frontal alpha suppression as a neural mechanism that may underlie leading behavior.

Although ML may be an appealing method to discover interaction markers in hyperscanning data, it nevertheless entails a number of challenges. First, while in the aforementioned study, leading behavior indices were picked up in nearly each pair, ML models are generally better at getting at group-level differences rather than being able to pick out individual-level neural indices (Davatzikos, 2019). Second, EEG data are quite noisy, and therefore applying ML to inter-brain data may be particularly prone to overfitting (Lever *et al*., 2016). Even models that are exceptional at data fitting, such as deep learning models, may not generalize well to new data sets (Davatzikos, 2019). Third, given that ML models are trained on large amounts of data, their complexity increases, and as a result their predictions and decisions are often difficult to interpret. Explainability methods offer promises in this regard, by e.g. looking at which features were important for prediction (Hansen and Rieger, 2019). However, the robustness of the findings when applying ML methods should always be carefully assessed (e.g. through cross-validation, Hosseini *et al*., 2020), and ideally tested in follow-up studies.

### What visualizations should I include when I am ready to present my results?

For intra-brain data, researchers have developed clear recommendations for ‘what’ to visualize (e.g. time-frequency data, scalp topographies, data distributions, metrics of variability, etc.) and ‘how’ to visualize them (e.g. recommendations for visual elements such as color schemes, axis labels, etc.; Keil *et al*., 2014, 2022). However, there are currently no established conventions for visualizing inter-brain data analyses or results. Accordingly, there is wide variability in how such analyses and results are presented across hyperscanning studies. Here, we propose a set of guidelines regarding what to visualize and discuss how different visualization options could be helpful for readers of hyperscanning studies. In essence, we recommend that researchers illustrate their preprocessing steps and show spatiotemporal visualizations of both intra-brain signals and inter-brain measures, in addition to showing aggregated statistical results.

#### Guideline 1: Visualize the pre-processing and statistical analysis pipeline with example data

Many hyperscanning studies feature highly complex pre-processing and statistical analysis pipelines, and the impact of each step on the data might not be immediately obvious. Providing an illustration of one’s pre-processing and analysis steps using example data would help to advance the field by allowing for much easier comparisons of methods between papers. Examples of visualizations of pre-processing steps that incorporate example data include Figure 2 from Gugnowska *et al*. (2022) and Figure 1 in Zamm *et al.* (2018) .

#### Guideline 2: Visualize intra-brain data checks with example data and grand averages

**Fig. 4.**
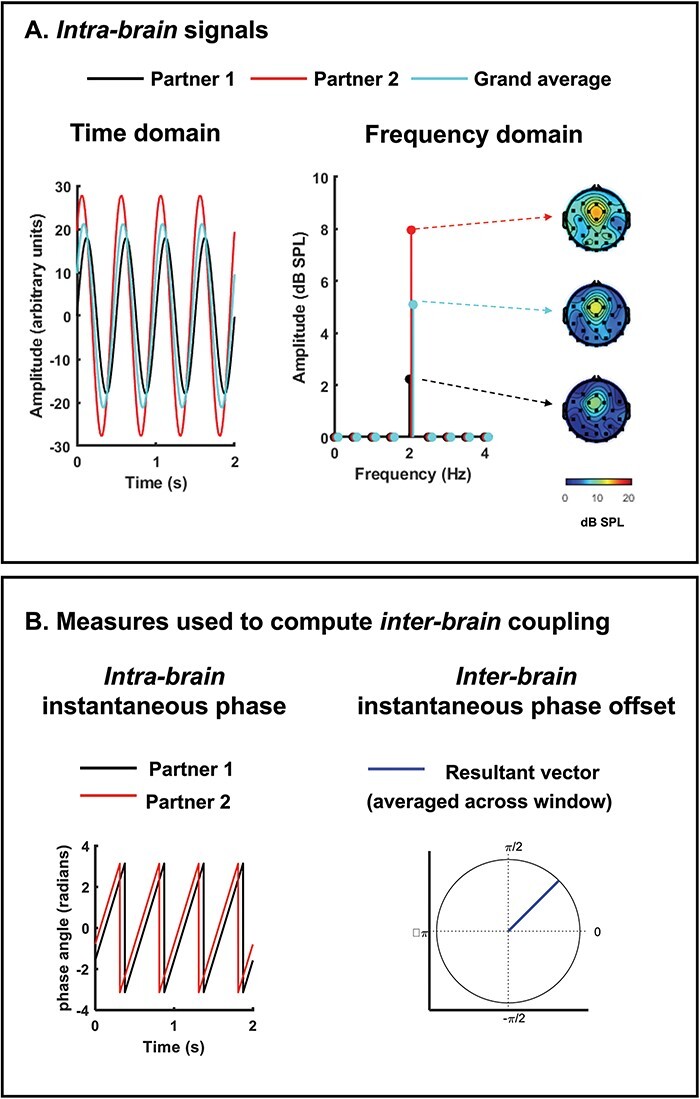
Simulated example of visualizing intra- and inter-brain signals (Guidelines 2–3). Visualizing intra-brain signals (Top panel): Simulated phase-shifted (*pi/*4) 2 Hz oscillations from two theoretical joint action partners (black and red lines) in the time-domain (left) and frequency-domain (right). Sinusoids are used to illustrate clear oscillatory signals and do not reflect properties of real EEG signals. Both partners clearly show spectral peaks in the frequency domain at 2 Hz. Topographies displaying spectral amplitude on each channel at the 2 Hz frequency indicate corresponding spatial patterns of EEG activity between partners, suggesting similar underlying neural processes. The grand average time- and frequency-domain signal reflects the similarity between partners’ EEG signals. Visualizing measures used to compute inter-brain coupling (Bottom panel): Example visualization for inter-brain phase-locking between partners. Bottom panel (left): Instantaneous phase for each partner (red and black lines) computed from their time-domain 2 Hz oscillations, for visual inspection of continuous phase offsets prior to computing inter-brain phase-locking. Bottom panel (right): Unit circle depicting inter-brain phase offset and phase-locking. The angle of the resultant vector represents the mean phase offset between partners and the vector length represents the degree of phase-locking. In this example, partners’ signals feature a constant phase relationship and therefore are fully phase-locked (corresponding to a PLV of 1).

It is beneficial to visualize the intra-brain data checks described in Section ‘What should I consider when initially inspecting the data?’ to demonstrate that signals of interest were identified in individual brains before intra- or inter-brain dependent measures were computed. Specifically, we suggest that researchers plot the time- and frequency-domain intra-brain signals from which dependent variables are computed, along with topographies of these signals, as illustrated in Figure 4A. Including grand average intra-brain signals along with example data from a small number of individual participants in published manuscripts can help reassure readers that the signal being analyzed reflects a brain response rather than an artifact and captures the oscillatory process of interest in the case that oscillations are being analyzed (e.g. alpha topographies reflecting maximal activity over central sites reflect different processes than topographies with maximal activity at occipital sites; see Section ‘What should I consider when initially inspecting the data?’ for further discussion).

#### Guideline 3: Visualize intra- and inter-brain dependent measures

The underlying intra-brain signals inspected in Guideline 2 (above) are used to derive intra-brain dependent measures (e.g. individual partners’ phase values), which in turn are used to compute inter-brain dependent measures (e.g. inter-brain phase-locking values). It is therefore important to visually inspect intra-brain dependent measures before computing inter-brain measures. We suggest that researchers visually compare partners’ intra-brain dependent measures within and across trials where possible. For example, if researchers are interested in computing phase-locking values between partners’ intra-brain phase values in a particular frequency band, they might plot partners’ intra-brain phase values in that frequency band overlaid on the same plot, as illustrated in Figure 4B (left panel). This would help to confirm that both partners’ intra-brain measures display minimal artifact and can help reassure readers that the data were not distorted by earlier pre-processing steps. The corresponding inter-brain measures can then also be plotted, as illustrated in Figure 4B (right panel), separately for each experimental condition.

#### Guideline 4: Provide spatiotemporal visualizations of key statistical findings

Finally, statistics computed on inter-brain measures should be displayed in both time and space if relevant. For example, a researcher comparing changes in inter-brain phase-locking across time-windows of musical performance could plot the time-course of statistical significance values, along with a topography of significance values for each time window (for example, see Figure 5 in Gugnowska *et al*., 2022). This can help readers assess the spatial distribution of statistical significance, ascertain what a given mean value indicates for a given analysis window and assess whether the statistic is likely to reflect coupling of true brain activity or noise (e.g. whether the spatial distribution of electrodes showing maximal inter-brain coupling display a coherent pattern, e.g. are clustered in regions associated with known neural phenomena).

#### Guideline 5: Provide your data visualization and analysis code online

We recommend that researchers provide clearly documented data analysis and visualization code alongside their published findings. This ensures that the published findings are reproducible and benefits future researchers who will face similar questions and challenges as they implement their own data processing, analysis and plotting pipelines. It is best practice that researchers plan to publish their code already at the first stages of data processing, facilitating their ability to create code and accompanying documentation that can be easily understood by future readers. Detailed guidelines for creating reproducible code are provided elsewhere (see, e.g.Botvinik-Nezer and Wager, 2022) and include recommendations such as documenting which type of environment the code runs in, indicating which versions of publicly available software packages or analysis scripts were used and creating meaningful variable and function names.

## Conclusion

The present article provides an overview of theoretical and practical points to consider when determining whether to use hyperscanning in a joint action research program. We provide a guide for newcomers to hyperscanning from various disciplines who seek to design experiments that investigate neural mechanisms of social coordination but are uncertain about where to begin. We underscore that there is much to gain from conducting hyperscanning studies, namely that researchers can identify inter-brain mechanisms of social interaction that cannot be elucidated from single-brain measurement alone, and we provide recommendations and suggestions to help researchers avoid the potential pitfalls of hyperscanning pointed out by skeptics. Specifically, we encourage researchers to start by carefully considering whether their research question requires hyperscanning or whether hyperscanning might instead add unnecessary complexity. Next, we encourage researchers to carefully design their studies with the goal of maximizing interpretability of inter-brain measures, such that it is clear whether they arise from endogenous *vs* exogenous sources. We describe how to carefully choose theoretically motivated dependent variables, including how to determine which of the common inter-brain measures to use, depending on the nature of one’s research question. Finally, we suggest numerous checks to implement during data analyses, most importantly checking the quality of intra-brain data before diving into inter-brain analyses. We also advocate for standardized visualization approaches (e.g. consistent plotting of intra- and inter-brain measures) to facilitate transparency and reproducibility.

Although there are existing review papers on joint action (Knoblich *et al*., 2011; Bolt and Loehr, 2021; Sebanz and Knoblich, 2021) and methods papers on technical implementation of hyperscanning set-ups and data analyses (Barraza *et al*., 2019; Ayrolles *et al*., 2021; Nguyen *et al*., 2021), acquiring knowledge of theory and methods through this existing literature may not be sufficient for newcomers to design hyperscanning studies that yield interpretable results. We hope that the present article fills this gap, by providing a step-by-step guide for how to design hyperscanning studies of joint action, putting researchers on more solid ground for making clear and valid insights into mechanisms underlying joint action and social interaction.

## Supplementary Material

nsae026_Supp
