# Supplementary material for: A practical guide to EEG hyperscanning in joint action research: from motivation to implementation
Source: Soc Cogn Affect Neurosci. 2024 Apr 3;19(1):nsae026. doi: 10.1093/scan/nsae026 (PMC11086947; doi:10.1093/scan/nsae026)
Supplement: nsae026_Supp [file nsae026_supp.zip › scan-23-106-File007.docx]

**Supplementary Material for the article**

**A Practical Guide to EEG Hyperscanning in Joint Action Research:**

**From Motivation to Implementation**

Anna Zamm^1,2*^, Janeen D. Loehr^3*^, Cordula Vesper^1,2^, Ivana Konvalinka^4^, Simon L. Kappel^5^, Ole A. Heggli^6^, Peter Vuust^6^, and Peter E. Keller^6,7^

^*^Shared first and corresponding authors.

^1^Department of Linguistics, Cognitive Science and Semiotics, Aarhus University, Denmark

^2^Interacting Minds Center, Aarhus University, Denmark

^3^Department of Psychology and Health Studies, University of Saskatchewan, Canada

^4^Section for Cognitive Systems, DTU Compute, Technical University of Denmark, Denmark

^5^Department of Electrical and Computer Engineering, Aarhus University, Denmark

^6^Center for Music in the Brain, Department of Clinical Medicine, Aarhus University & The Royal Academy of Music Aarhus/Aalborg, Denmark

^7^MARCS Institute for Brain, Behaviour and Development, Western Sydney University, Australia

## ***S1*** ***How is phase synchrony measured?***

A common approach to measuring phase synchrony is to extract the instantaneous phase of the waveform in question at each point in a time-window of interest (e.g., Bruns & Eckhorn, 2004), and then compute either the Phase Locking Value (PLV; (Lachaux et al., 1999), the Phase Lag Index (PLI; (Stam, Nolte, & Daffertshofer, 2007), or the Circular Correlation Coefficient (CCorr; (Burgess, 2013; Jammalamadaka & Sengupta, 2001) between the two waveforms’ instantaneous phases (also referred to as phase coherence; (Mormann, Lehnertz, David, & Elger, 2000).

PLV is computed by (1) taking the instantaneous phase difference between two waveforms at selected timepoints, (2) projecting them onto a unit circle, and (3) computing the mean vector length across phase angles, as illustrated in the unit circle plots in the bottom panel of Figure 3A in the main text. The mean vector length is defined as the PLV, and reflects the circular variance of phase differences. PLVs range from 0 to 1, where 0 indicates random phase offsets between waveforms (uniform distribution of phase differences) and 1 indicates a perfect phase-locking between waveforms (non-uniform distribution). Lachaux et al. (1999) provide recommendations for how to assess chance-levels of phase-locking using phase-locking statistics (PLS). PLVs have been used in foundational EEG hyperscanning studies (Dumas et al., 2011; Dumas et al., 2010), and notably, phase-locking is used as a measure of interpersonal action synchrony in studies that solely examine behaviour (e.g., Sabharwal et al., 2022; Tomassini, 2022), and as a measure of physiological synchrony in studies that measure signals such as respiration and heart rates (Konvalinka, Sebanz, & Knoblich, 2023).

Stam et al. (2007) proposed the Phase Lag Index (PLI) as an alternative metric to the PLV, because spuriously high PLVs can arise in EEG and MEG measurement from volume conduction or shared noise between electrodes (Nolte et al., 2004). The PLI has been widely adopted by the hyperscanning community (e.g., Ahn et al., 2018; see Czeszumski et al., 2020 for further references). The PLI is similar to the PLV in that it assesses the circular variance of phase angle differences between waveforms, but mathematically excludes phase locking values that are uniformly distributed around 0 and 180, as these are likely to arise from shared neural or noise sources (Stam et al., 2007). The PLI also ranges from 0 to 1, where 0 indicates no coupling or spurious coupling (arising from shared sources / noise) and 1 indicates phase-locking between waveforms. That said, volume conduction should not impact inter-brain PLV measures computed across corresponding electrodes / regions of interest on different scalps, so the PLI is not necessarily advantageous in the case of inter-brain measurement unless there are suspected sources of shared noise.

Newcomers to EEG hyperscanning might be confused by the fact that there are some inconsistencies with regards to how the terms PLV, PLI, and phase coherence are used across the literature. For example, in an influential study of intra- and inter-brain phase-locking between guitar duettists, Sänger, Müller, and Lindenberger (2012) refer to inter-brain PLV as phase coherence and use the term PLV to reference intra-brain measures of phase consistency at specific sensorimotor electrode sites across trials in different frequency bands. The reason for variations in terminology is likely that PLV and PLI originated as single-subject measures of phase locking between electrodes and a stimulus. Notably, in single-subject studies, PLV and PLI typically compare phase locking to a stimulus across trials at specific time-windows of interest. In contrast, hyperscanning studies often assess phase dynamics across successive time-points within trials of a joint task (rather than across trials; see Burgess, 2013, for further comparison of PLV definitions). A straightforward way to minimize confusion and help facilitate a standard descriptive language is to use the term *inter-brain PLV / PLI* when describing phase-locking measures computed between brains, and to be clear about whether the PLV / PLI metrics are computed across trials or over time within-trials, as the choice to compute within- versus across-trial phase-locking can have critical implications for interpretation.

An alternative to the PLV and PLI for measuring inter-brain phase synchrony is the Circular Correlation Coefficient (CCorr; (Burgess, 2013; Jammalamadaka & Sengupta, 2001), which quantifies the degree to which two oscillatory waveforms co-vary from their predicted phase over time. The logic underlying this measure is that the expected phase of an ideal oscillator can be computed for any point in its cycle; because real-world oscillators are never ideal (they are susceptible to random and non-random fluctuations), their variance from the expected phase of an ideal oscillator at the same frequency can be computed. If two real-world oscillatory waveforms are synchronized, then their phase deviations should covary over time. Burgess (2013) provides compelling evidence for the robustness of CCorr over PLV for capturing synchronous phase dynamics over time. Specifically, the PLV can be high (i.e., show consistently small phase differences) between signals that have identical frequencies but do not mutually influence one another (or are not mutually influenced by a third variable). However, a different formula has been proposed for CCorr which may be more appropriate when dealing with continuous signals (see Zimmermann et al., in prep); however, while leading to more robust estimates of CCorr, they are also more comparable to PLVs.

## ***S2*** ***How is envelope coupling measured?***

Envelope coupling is typically measured by first (1) narrow-band filtering two waveforms in a frequency band of interest to extract specific oscillations, (2) computing the Hilbert transform of each oscillation (otherwise known as the “analytic signal”, the magnitude of which corresponds to the signal envelope), (3) extracting the real-valued envelope of the Hilbert transform, (4) and, finally, correlating the resulting envelopes using a Fisher-transformed Pearson correlation (Brookes et al., 2011; Bruns & Eckhorn, 2004; Hipp et al., 2012).

## ***S3*** ***How is wavelet coherence measured?***

Wavelet coherence is typically measured by (1) convolving individual waveforms with wavelets (short oscillatory signals with amplitude ranging from 0 to some maximum and then back to 0; Cohen, 2014; Hu et al., 2021; Nguyen, Hoehl, & Vrticka, 2021) across all the relevant EEG frequencies in some selected range to obtain an estimate of the spectral coherence between the signal and the wavelet at each frequency, and then (2) comparing the wavelet coherence time-series of two signals to obtain an estimate of whether they display correlated spectral change over time.

## ***S4 How is Partial Directed Coherence measured?***

First, spatial locations and frequencies of interest should be defined for where directional influences are expected based on *a priori* hypotheses (see Section 4.2 in the main text for further discussion). A multivariate autoregressive (MVAR) model is fitted to the EEG time-series, which aims to predict the data at each sample from a linear combination of a number (defined as the model order) of previous samples (Hoerzer, Liebe, Schloegl, Logothetis, & Rainer, 2010). Once the MVAR model is estimated, it is transformed to the frequency domain by applying a Fourier transform to the model. PDC is then computed for each pair of ROIs *i* and *j* (from person A and person B and vice-versa), and normalized in the range of [0,1], with larger values indicating stronger influence (Astolfi et al., 2010). PDC thus measures the directional flow of information directly from ROI *j* to ROI *i* at a frequency *f*, with respect to the total influence of *j* on all other ROIs (Baccalá & Sameshima, 2001).
